# Genomic profile and infection dynamics of Kashi_RDG1 (KRDG1), a novel cluster K1 mycobacteriophage infecting mycobacterial hosts

**DOI:** 10.3389/fmicb.2026.1859270

**Published:** 2026-07-13

**Authors:** Anuja Kakkar, Garima Kandwal, Tanmayee Nayak, Lav Kumar Jaiswal, Ankush Gupta

**Affiliations:** Molecular Microbiology Laboratory, Department of Biochemistry, Institute of Science, Banaras Hindu University, Varanasi, Uttar Pradesh, India

**Keywords:** antibiotic resistance, cluster K1 mycobacteriophage, KRDG1, multiplicity of infection, *Mycobacterium tuberculosis*, phage therapy, temperate phage

## Abstract

**Introduction:**

Tuberculosis (TB), caused by *Mycobacterium tuberculosis*, remains a major global health challenge, particularly due to the increasing emergence of multidrug-resistant strains and limited treatment options. Bacteriophages have gained attention as potential alternatives or adjuncts to conventional antibiotics owing to their host specificity and antibacterial efficacy.

**Methods:**

This study reports the isolation and detailed characterization of mycobacteriophage Kashi_RDG1 (KRDG1), isolated using *Mycobacterium smegmatis* mc^2^155. Genomic analysis, transmission electron microscopy, host range analysis, one-step growth assay, adsorption assay, multiplicity of infection (MOI) determination, and infection kinetics were performed to characterize the phage.

**Results:**

Genomic analysis identified KRDG1 as sub cluster K1 mycobacteriophage, with a genome size of 58,681 bp containing 95 predicted open reading frames (ORFs), of which 39 are functionally annotated. Transmission electron microscopy analysis confirmed its siphovirus-like morphology while host range analysis depicted its polyvalent activity against *Mycobacterium fortuitum* (opportunistic pathogen) and *M. tuberculosis* H37Ra (an attenuated *Mtb* strain) in addition to *M. smegmatis*. One-step growth analysis revealed latent period of 80 min and burst size of 100 phage/bacterial cell supporting its efficient infection dynamics. Notably, infection kinetics demonstrated strong host bacterial killing during the logarithmic phase.

**Discussion:**

While KRDG1 exhibits temperate characteristics, its close genomic similarity to previously engineered therapeutic phage (ZoeJ) highlights its potential for future genetic engineering and therapeutic exploration against pathogenic mycobacterial and non-mycobacterial infections.

## Highlights

- KRDG1 is a novel mycobacteriophage belonging to the therapeutically relevant sub cluster K1.
- Genome of 58,681 bp with 95 ORFs; 39 ORFs have assigned functions.
- Polyvalent; demonstrates infectivity against *M. smegmatis, M. fortuitum* and *M. tuberculosis* H37Ra strains
- Depicts 80 min latent period, ~100 burst size, and efficient host lysis during log phase.
- Temperate nature with therapeutic potential through genetic engineering.

## 1 Introduction

Tuberculosis (TB) is a chronic granulomatous disease caused by *Mycobacterium tuberculosis (Mtb)*, a slow-growing, intracellular pathogen with a lipid-rich, mycolic acid-containing cell wall that contributes to its persistence and drug resistance (Ghazaei, 2018). In addition to *Mtb*, the genus *Mycobacterium* includes a range of nontuberculous mycobacteria (NTM) that can cause opportunistic infections, particularly in immunocompromised individuals. While TB accounts for the vast majority of mycobacterial disease burden worldwide, NTM infections are increasingly recognized in clinical settings and often exhibit intrinsic resistance to multiple antibiotics (Ratnatunga et al., 2020). Among these, *Mycobacterium fortuitum* is a rapidly growing opportunistic pathogen commonly found in environmental sources such as soil and water. *M. fortuitum* can cause clinically significant infections including pulmonary disease, skin and soft tissue infections, post-surgical wound infections, catheter-associated infections, and prosthetic device-related infections, particularly in individuals with preexisting lung conditions. Its biofilm-forming ability and variable antimicrobial susceptibility further complicate treatment, highlighting the need for alternative therapeutic approaches (Kim et al., 2023).

In 2023, TB affected more than 10.8 million individuals and caused approximately 1.25 million deaths, making it the world’s deadliest bacterial infection (World Health Organization, 2024). The burden remains concentrated in low- and middle-income countries, particularly in the South and Southeast Asia and across Africa. India alone accounted for 26% of global TB cases and 29% of TB-related deaths among HIV-negative individuals. Alarmingly, an estimated 400,000 individuals developed multidrug-resistant or rifampicin-resistant tuberculosis (MDR/RR-TB), representing a major challenge to TB control efforts. MDR-TB, defined by resistance to both rifampicin the most potent first-line anti-TB drug and isoniazid, requires prolonged treatment with second-line drugs that are less effective, more toxic, and significantly costlier. India alone accounted for 27% of global MDR/RR-TB cases, making it a key priority in global TB intervention strategies. The overall burden of MDR/RR-TB remains a serious public health threat (World Health Organization, 2024).

Although emerging approaches such as host-directed therapies and CRISPR-based antimycobacterial strategies offer mechanistic rationale for combating drug-resistant infections, their clinical translation remains limited due to challenges in *in vivo* delivery, potential off-target effects, and suboptimal efficacy against persistent pathogen (Modell et al., 2022). Whereas these approaches remain largely experimental, bacteriophages represent a biologically precise, self-replicating antimicrobial alternative with proven activity against pathogenic bacteria. By recognizing specific bacterial strains, phages infect, replicate, and lyse their hosts, releasing progeny virions that sustain the therapeutic effect. Their efficacy remains unaffected by conventional antibiotic resistance, and their targeted action may reduce disruption of commensal microbiota compared to broad-spectrum antibiotics (Kutter and Sulakvelidze, 2004). Decades of clinical use in countries like Georgia, Russia, and Poland further support the safety and therapeutic value of phage therapy, supporting its potential application in antimicrobial strategies (Venturini et al., 2022).

Mycobacteriophages are tailed double-stranded DNA viruses that specifically infect *Mycobacterium* species. They belong to the class Caudoviricetes and display considerable genetic diversity. Based on nucleotide similarity, these phages are grouped into 29 clusters (A–Z, AA–AC) and 10 singletons, with members of a cluster sharing more than 35% sequence identity. Most mycobacteriophages have been isolated using *Mycobacterium smegmatis* mc^2^155, though a few infect *Mtb* directly. Their ability to breach the complex, lipid-rich mycobacterial cell wall makes them valuable tools for both diagnostics and therapeutics (Hatfull, 2020). Mycobacteriophages exhibit lytic or temperate life cycles (Nayak et al., 2019), regulated by early and late gene expression. Early genes are involved in regulation, while late genes (~30 min post-infection) drive virion assembly and host lysis, typically completed within 180 min (Hatfull, 2018).

Mycobacteriophages have proven valuable in diagnostics with reporter phages engineered to deliver fluorescent or selectable markers enabling rapid detection and drug susceptibility profiling (Piuri and Hatfull, 2018). Capture systems utilizing whole phages or phage-derived proteins have also been employed to isolate target mycobacteria from complex biological samples (Arutyunov et al., 2014; Hosseiniporgham and Sechi, 2022). Therapeutically, engineered phages have demonstrated efficacy in treating refractory nontuberculous mycobacterial infections. A personalized three-phage cocktail achieved clinical resolution of disseminated *Mycobacterium abscessus* in a cystic fibrosis patient following lung transplantation (Dedrick et al., 2019a). Similarly, adjunctive phage therapy targeting *Mycobacterium chelonae* led to sustained improvement in an immunosuppressed patient, even in the presence of neutralizing antibodies (Little et al., 2022). These studies demonstrate the applicability of mycobacteriophages in difficult-to-treat mycobacterial infections. One such group of therapeutically relevant mycobacteriophages belongs to Cluster K, which are known to infect clinically significant *Mycobacterium* species. This cluster comprises both lytic phages (TM4-derived from a temperate ancestor) and temperate phages (ZoeJ, Fionnbharth, and Adephagia). However, because temperate phages can integrate into the host genome and potentially carry genes associated with lysogeny or horizontal gene transfer, they are not directly suitable for therapeutic use. To overcome this, genetic engineering strategies such as BRED (Bacteriophage Recombineering of Electroporated DNA) have been employed to delete lysogeny-associated genes, including integrase and repressor genes, thereby converting them into lytic derivatives capable of safe and effective therapeutic applications (Hatfull, 2018; Kakkar et al., 2024).

In view of the growing threat of antimicrobial resistance in pathogenic *Mycobacterium* species, there is an urgent need to identify, characterize and develop a repertoire of bacteriophages that can be used for diagnostic and therapeutic applications. While 2,799 mycobacteriophage genomes have been sequenced and deposited in the PhagesDB database^1^ (accessed May 2026), detailed functional characterization has been performed for only a limited subset. To date, ∼18 scientific reports have described the functional characterization of mycobacteriophages, and among them, only a few have investigated key experimental parameters such as optimal multiplicity of infection (MOI), latent period, burst size, thermal and pH stabilities, all of which are critical for evaluating their potential in therapeutic applications (Nayak et al., 2025; Nayak et al., 2023; Zaychikova et al., 2025; Guo et al., 2023; Lang et al., 2023; Lopez-Davis et al., 2022; Gan et al., 2014; Sinha et al., 2020; Reddy and Gopinathan, 1987; Fan et al., 2015; Gong et al., 2021; Basra et al., 2014; Harman-McKenna and De Buck, 2023; Das et al., 2024; Stella et al., 2013; Schäfer et al., 1977; David et al., 1980; Li et al., 2024). To address this gap, we report the isolation and comprehensive characterization of a novel mycobacteriophage, KRDG1, using *M. smegmatis* as the host. We investigate its morphology, genomic architecture, comparative genomic relationships, infection kinetics, and host range, providing insight into its distinct biological and genomic features and potential utility in advancing phage-based strategies targeting pathogenic *Mycobacterium* species.

## 2 Materials and method

### 2.1 Bacterial hosts and culture protocols

*M. smegmatis* (ATCC 700084), the primary host for phage isolation and characterization, was cultured on LB agar (1.5% w/v) supplemented with 0.4% (v/v) glycerol and 0.1% Tween-80 (LBGT medium) at 37 °C. Liquid cultures were grown in LBGT at 150 rpm until mid-log phase (Optical density (OD₆₀₀) ≈ 0.4–0.5). For phage isolation and propagation, Tween-80 was removed by centrifugation (2000 × g, 5 min) and resuspension in fresh LB medium without Tween. For plaque assays, double-layer agar plates were prepared using 1.5% LBG agar as bottom and 0.8% LBG agar as top layers, supplemented with 1 mM CaCl_₂_. *M. fortuitum* (opportunistic pathogen) was grown in LBGT medium, while *Mtb* H37Ra (an attenuated *Mtb* strain) and *Mycobacterium marinum* were cultured in Middlebrook 7H9 broth containing 0.2% glycerol and 10% OADC supplement, and plated on Middlebrook 7H10 agar with 0.5% glycerol and 10% OADC.

Non-mycobacterial strains like *Enterococcus faecalis, Staphylococcus aureus, Escherichia coli* DH5α, *Klebsiella pneumoniae* and *Pseudomonas aeruginosa* were cultured on LB broth and agar under standard aerobic conditions at 37 °C with shaking at 150 rpm. All bacterial work was conducted in accordance with standard BSL-2 biosafety guidelines (Zaychikova et al., 2025).

### 2.2 Phage isolation and propagation

Environmental soil samples (*n* = 48) were collected from various locations across the Banaras Hindu University (BHU) campus, Varanasi, India. Approximately 2–3 g of each sample was suspended in 3 mL of sterile phage buffer [10 mM MgSO₄, 1 mM CaCl_₂_,10 mM Tris–HCl (pH 7.5) and 68.5 mM NaCl] (PhagesDB.org) and supplemented with 0.5 mL of *M. smegmatis* culture grown to mid-log phase. The mixtures were incubated overnight at 37 °C with shaking at 150 rpm to enrich for phages. Following incubation, the samples were centrifuged at 8000 × g for 5 min and the supernatants were filtered through 0.22 μm syringe filters to remove residual bacteria. The resulting filtrates were spotted directly onto lawns of *M. smegmatis* prepared on double-layer LBG agar plates. Plates were incubated at 37 °C for 24–48 h and examined for plaque formation. Plaques observed on the bacterial lawn were subsequently picked and purified for further characterization (Hyman, 2019).

### 2.3 Phage morphology analysis via TEM

The morphology of the isolated mycobacteriophage was examined using transmission electron microscopy (TEM). A 10 μL aliquot of high-titer phage suspension (~10^8^ PFU/mL) was applied on a carbon-coated copper grid and allowed to adsorb for 1 min. Excess liquid was carefully removed using filter paper, followed by a brief rinse with sterile distilled water. The grid was negatively stained with 1% phosphotungstic acid (PTA, pH ~ 7.0) for 1 min and subsequently air-dried. Imaging was performed using a TECNAI 200 kV TEM (SAIF Facility, AIIMS, New Delhi) to visualize virion morphology (Ackermann, 2009).

### 2.4 Genomic DNA isolation, sequencing, and annotation

Mycobacteriophage KRDG1 lysate was filtered through a 0.22-μm syringe filter, and genomic DNA was extracted using the phenol-chloroform-isoamyl alcohol (25:24:1)–SDS method as described in the PhagesDB database. Briefly, 1 mL of filtered lysate was treated with DNase I (0.1 U/mL) and RNase A (4 μg/mL) at 37 °C for 90 min to degrade host nucleic acids, followed by incubation with SDS, EDTA and proteinase K at 55 °C for 1 h. DNA was extracted with PCI, precipitated using sodium acetate and ethanol, washed with 70% ethanol, and resuspended in deionized water (Nishiguchi et al., 2002).

Whole-genome sequencing of KRDG1 was performed by MedGenome (Bengaluru, India) using Illumina paired-end Next-Generation Sequencing technology, generating paired-end FASTQ files (R1 and R2). Read quality was assessed using FastQC on the CPT-GALAXY platform^2^. Random subsampling was performed at 50,000 reads prior to genome assembly. *De novo* genome assembly was carried out using SPAdes with default parameters, including the unpaired/single-read option. Multiple contigs were generated, and the contig with the highest coverage was selected as the final genome assembly. Genome termini and packaging strategy were determined using PhageTerm. Genome annotation was performed using the PECAAN (Phage Evidence Collection and Annotation Network) pipeline. The assembled FASTA genome sequence was initially analyzed using GeneMark.hmm for host-trained coding potential prediction and subsequently imported into PECAAN for manual annotation. Open reading frames (ORFs) predicted by Glimmer and GeneMark were manually curated based on coding potential, gene length, overlap/gap size, ribosome binding site (RBS) z-score, and spacer length. ORFs ≥100 bp with minimal overlaps or gaps were prioritized during annotation. Functional annotation was assigned based on sequence homology and conserved domain analyses using NCBI BLASTp, HHpred, and the Conserved Domain Database (CDD), while genes lacking significant functional predictions were annotated as hypothetical proteins/no known function (NKF). Transmembrane domains were analyzed using TMHMM, and the presence of tRNA and tmRNA genes was also examined. Promoter regions were predicted using the PhagePromoter tool (Neural Network Promoter Prediction), transcription terminators were identified using the ARNold server, and a circular genome map was generated using the CGView tool (Grant et al., 2023).

#### 2.4.1 Sequence clustering and comparative genomic analysis

Sequence clustering and comparative genomic analysis of mycobacteriophage KRDG1 were conducted using the Phagescope platform^3^. Accordingly, the study was conducted in two stages: (i) comparative clustering among closely related subcluster K1 phages based on gene-content similarity, and (ii) comparative analysis with representative sub cluster K2 and K4 phages to evaluate broader genomic differences across mycobacteriophage groups.

All phages analyzed belong to the class Caudoviricetes under the ICTV framework. For sequence clustering, 19 closely related K1 subcluster phages were selected based on BLASTn similarity (≥90% query coverage and ≥92.7% identity). In addition, average nucleotide identity (ANI) analysis was performed using the EzBioCloud ANI calculator to further validate genome-level similarity among the selected sub cluster K1 phages^4^. Clustering was then performed using MMseqs2-based hierarchical analysis of shared gene content.

Comparative sequence alignment was carried out using BLASTP of annotated protein sequences within PhageScope to assess protein-level similarity across key functional genes. Two comparative genomic alignments were generated: (i) KRDG1 (K1), ZoeJ (K2), and Fionnbharth (K4) to evaluate broader genomic differences across Cluster K subclusters and identify conserved gene modules, syntenic regions, and variable genomic regions, and (ii) KRDG1 (K1), Kashi_VT1 (K1), and CrimD (K1) to examine genomic relationships within the K1 subcluster.

### 2.5 Thermal and pH stability test

The thermal stability of mycobacteriophage KRDG1 was assessed by incubating approximately 10^8^ PFU/mL at a range of temperatures (4 °C, 15 °C, 25 °C, 37 °C, 45 °C, 55 °C, and 65 °C) for 1 h. Following incubation, the residual phage titers were determined using the double-layer agar method. For pH stability, 100 μL of KRDG1 phage suspension (∼10^8^ PFU/mL) was mixed with 900 μL of saline solution adjusted to different pH values ranging from 3.0 to 11.0. Saline-based pH-adjusted solutions were used due to precipitation observed during alkaline adjustment of the standard phage buffer. The mixtures were incubated at room temperature for 1 h. After incubation, the remaining phage titers were quantified using the double-layer agar method. To assess the effect of buffer composition, phage titers were determined in saline and phage buffer at pH 7 and used to calculate the efficiency of plating (EOP). Both experiments were performed in triplicate (Taj et al., 2014).

### 2.6 Host range

Host range analysis was performed on three mycobacterial strains- *M. fortuitum* (ATCC 6841), *Mtb* H37Ra (ATCC 25177), and *M. marinum* (ATCC 927) and five non-mycobacterial strains – *E. coli* (DH5alpha) *E. faecalis* (ATCC 19433), *P. aeruginosa* (ATCC 27853), *S. aureus* (ATCC 12600), and *K. pneumoniae* (ATCC 13883). For each strain, 0.5 mL bacterial culture was mixed with 4.5 mL molten top agar (0.8% agar) and overlaid onto agar plates. Serial dilutions of phage KRDG1 (10^−1^to 10^−8^) were prepared in phage buffer, and 3 μL of each dilution was spotted onto the bacterial lawns. LB plates were used for all strains except *Mtb* H37Ra and *M. marinum* for which 7H10 agar plates were used. Plates were incubated at 37 °C for 24 h for *M. fortuitum* and all non-mycobacterial strains, and for 7–14 days for *M. marinum* and *Mtb* H37Ra. Plaque formation was used to determine the host range (Zaychikova et al., 2025).

### 2.7 Determination of optimal multiplicity of infection (MOI)

*M. smegmatis* cultures were grown to mid-log phase (OD₆₀₀ ≈ 0.5–0.6), corresponding to approximately 5 × 10^7^ CFU/mL. The bacterial cultures were diluted and infected with phage KRDG1 at varying MOIs: 0.00001, 0.0001, 0.001, 0.01, 0.1, 1, 10, and 100. Infected cultures were incubated at 37 °C with shaking at 150 rpm for 6 h. Following incubation, samples were centrifuged at 5,000 × g for 3 min to pellet bacterial cells, and the supernatant was filtered through a 0.22 μm syringe filter to remove any residual bacteria. Phage titers (PFU/mL) were determined using the double agar overlay method. MOI producing the highest phage titer was considered to be the optimal MOI. The experiment was performed in triplicate (Guo et al., 2023).

### 2.8 One step growth curve

One-step growth curve is performed to determine the latent period and burst size of the phage. *M. smegmatis* cultures in mid-log phase (OD₆₀₀-0.4–0.5) were infected with phage at a MOI of 0.0001 and incubated at 37 °C for 30 min with shaking to facilitate adsorption. Following incubation, cells were pelleted by centrifugation at 5,000 × g for 5 min to remove unadsorbed phages and the pellet was resuspended in fresh LBG medium. The culture was further incubated at 37 °C and samples were collected every 20 min for up to 180 min. Phage titers at each time point were determined using the double agar overlay method. The experiment was performed in triplicate (Ellis and Delbruck, 1939).

### 2.9 Adsorption curve assay

*M. smegmatis* cells with mid-log phase were mixed with phage KRDG1 at a MOI of 0.0001 in LBG medium and incubated at 37 °C with shaking at 150 rpm. At 10-min intervals over a 90-min period, samples were centrifuged at 5,000 × g for 3 min, and 50 μL aliquots of the supernatant were collected. Each aliquot was used to determine PFU by the double-layer agar method. The experiment was performed in triplicate (Zaychikova et al., 2025).

### 2.10 Infection kinetics of *M. smegmatis* in response to phage KRDG1 and antibiotics

The infection kinetics of *M. smegmatis* were assessed during the lag and logarithmic (log) phases with the phage KRDG1 and the antibiotics isoniazid and rifampicin. For this assay, 25 mL of LBGT medium was inoculated with 1% (v/v) of a primary culture in the log phase (OD₆₀₀ ≈ 1.0) and incubated at 37 °C with shaking at 150 rpm. OD₆₀₀ was monitored, and upon reaching the lag phase (OD₆₀₀ ≈ 0.18) and log phase (OD₆₀₀ ≈ 0.5), cultures were treated with antibiotics isoniazid (100 μg/mL) and rifampicin (40 μg/mL). Although a low MOI was optimal for phage amplification in plate-based assays, but infection kinetics performed in liquid culture requires higher phage inputs to ensure effective infection and bacterial growth suppression due to differences in infection dynamics, host density, and phage-host interactions. Therefore, phage KRDG1 was applied at an MOI of 1 for lag-phase cultures and an MOI of 5 for log-phase cultures (Abedon, 2016). OD₆₀₀ readings were recorded at regular time intervals to monitor bacterial growth to evaluate and compare the individual effects of phage and antibiotics on bacterial viability. This experiment was performed in triplicate as per Kalapala et al. (2020).

### 2.11 Fluorescence microscopy

At OD₆₀₀ 0.5, *M. smegmatis* culture was infected with mycobacteriophage KRDG1 at an MOI of 5 and incubated at 37 °C with shaking at 150 rpm. Samples were collected at 1 h, 2 h, 3 h, and 4 h post-infection and 10 μL of sample was taken at each time point for analysis. Each sample was stained with Nile Red (final concentration: 4 μg/mL) and DAPI (final concentration: 6 μg/mL) for 5–10 min in the dark to visualize lipid membranes and nucleic acids, respectively. The samples were then spotted onto pre-formed 1% low-melting agarose pads (prepared in Milli-Q water) placed on notch glass slides. Imaging was performed using Nikon 90i fluorescence microscope with a 60 × objective lens and appropriate DAPI/TRITC filter sets, and images were processed using ImageJ. JS software (version 1.53 m). The image is representative of triplicate experiments (Thammatinna et al., 2020).

### 2.12 Statistical analysis

All graphs were generated using GraphPad Prism software (version 5.01). Thermal and pH stability data for phage KRDG1 were analyzed using Dunnett’s multiple comparisons test, while the infection kinetics of *M. smegmatis* (KRDG1 vs. antibiotics) were evaluated using Tukey’s multiple comparisons test. Results are expressed as mean ± standard deviation from three independent experiments.

## 3 Results

### 3.1 Isolation and plaque morphology of novel mycobacteriophage

**Figure 1:**
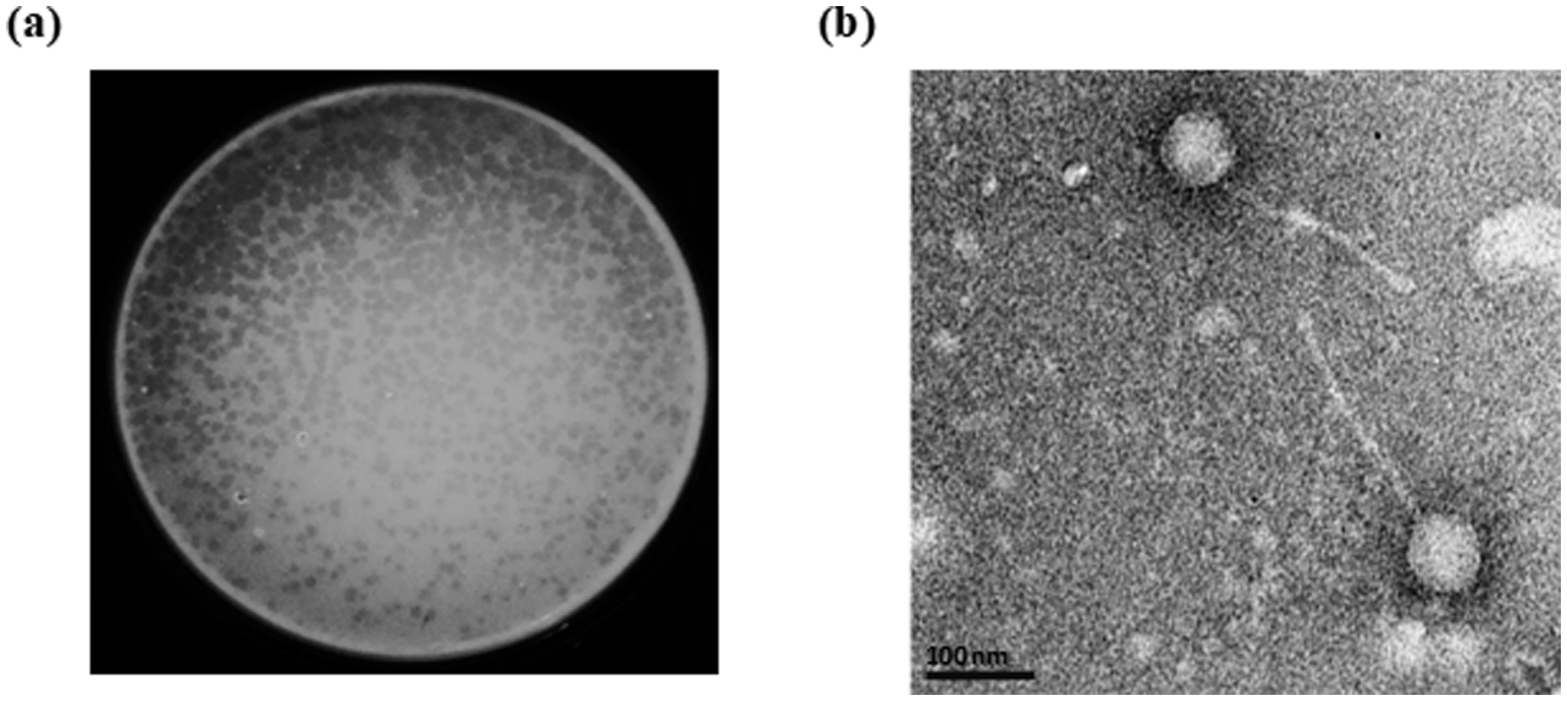
Morphological characterization of mycobacteriophage KRDG1. **(a)** Representative plaques formed by KRDG1 on a *Mycobacterium smegmatis* mc^2^155 lawn after 24 h of incubation at 37 °C. The average plaque diameter was approximately 1.45 ± 0.37 mm. **(b)** Transmission electron micrograph (TEM) of phage KRDG1, imaged after negative staining with 1% phosphotungstic acid. The phage displays a typical Siphoviridae morphology, with a total length of 189.94 ± 49.93 nm, a head diameter of 49.85 ± 11.89 nm, and a tail length of 141.23 ± 36.48 nm.

Mycobacteriophage KRDG1 was isolated using *M. smegmatis* as the host organism through double layer agar technique. The phage formed clear plaques with an average diameter of 1.45 ± 0.37 mm (Figure 1a). The phage was named KRDG1, with K denoting Kashi, the traditional name of Varanasi, and RDG referring to Ravidas Ghat, the location from which the environmental sample was obtained. Geographical coordinates of Ravidas Ghat, Varanasi are 25.28360 N 83.0092 E.

### 3.2 Phage morphology analysis *via* TEM

TEM of mycobacteriophage KRDG1, negatively stained with 1% phosphotungstic acid (PTA), revealed a head diameter of 49.85 ± 11.89 nm and a long, flexible, non-contractile tail measuring 141.23 ± 36.48 nm, with a total length of 189.94 ± 49.93 nm (Figure 1b). Based on these morphological features, KRDG1 exhibits siphovirus-like morphology.

### 3.3 Genomic features of KRDG1

**Figure 2:**
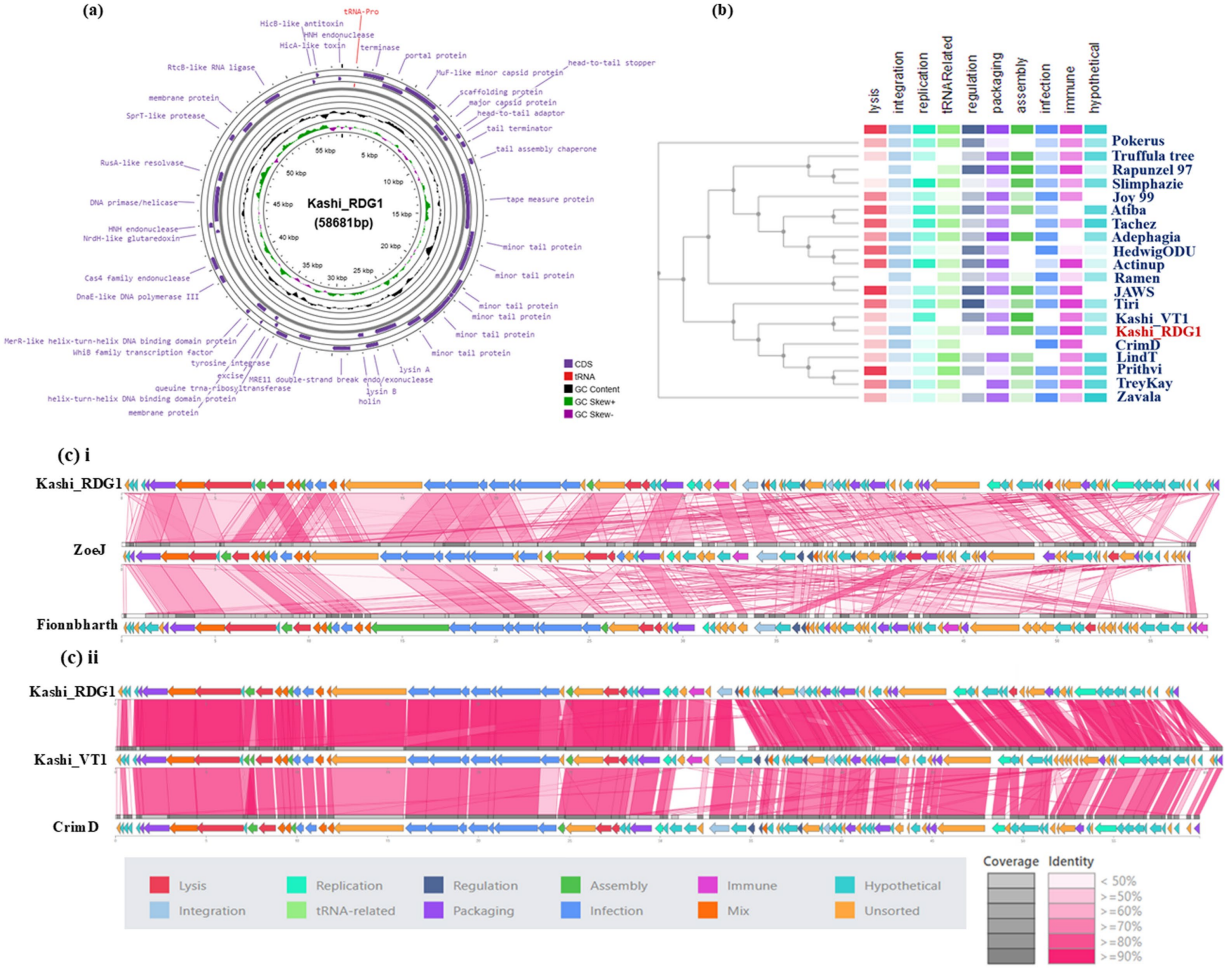
**(a)** Complete genome of phage KRDG1 consisting of 58,681 bp. Visualization of the circular genome map was performed using the CGView tool (http://stothard.afns.ualberta.ca/cgview_server/). Predicted ORFs/annotated genes, tRNA sequences, and GC content are shown in purple, red, and black, respectively. ORFs in the forward and reverse orientations are represented in the outer and inner rings, respectively. Reverse-strand ORFs were annotated as hypothetical proteins/no known function (NKF) and are therefore not displayed. **(b)** Functional gene-based clustering (synteny) of mycobacteriophage KRDG1 using PhageScope. Clustering was performed based on gene content across key functional categories, including lysis, integration, replication, tRNA-related genes, regulation, packaging, assembly, infection, immunity, and hypothetical proteins. Each row corresponds to an individual phage, and each column represents a functional gene annotation category. The clustering on the left indicates hierarchical grouping based on the presence or absence of genes and shared gene-content similarity. KRDG1 is highlighted in red and exhibits the greatest gene-content similarity with Kashi_VT1 and CrimD. All analyzed phages belong to Cluster K1 mycobacteriophages within the class Caudoviricetes. **(c)** Comparative genomic alignment of KRDG1 with representative Cluster K mycobacteriophages. (i) Comparative genomic alignment of KRDG1 (subcluster K1) with therapeutically investigated phages from other subclusters; like ZoeJ (subcluster K2) and Fionnbharth (subcluster K4). (ii) Comparative genomic alignment of KRDG1 (subcluster K1) with the closely related subcluster K1 phages like Kashi_VT1 and CrimD. Linear genome maps are displayed with genes represented as arrows indicating orientation and predicted function, color-coded according to annotation category. Regions of protein-level similarity were visualized using BLASTP of annotated protein sequences, with shaded bands representing pairwise protein sequence identity ranging from ≥50% to ≥90% as indicated by the gradient scale. Genomes are aligned and scaled according to genome length (kb).

The complete genome of KRDG1 is 58,681 base pairs in length, with an average coverage of 108.05 × and a G + C content of 66.6% (Figure 2a). KRDG1’s complete genome sequence has been submitted to the NCBI GenBank database under accession number ON687736.1. Phage terminal analysis using PhageTerm revealed fixed termini and a cos-type DNA packaging mechanism. Based on whole-genome BLASTn analysis, KRDG1 is most closely related to mycobacteriophage KVT1, exhibiting 99.50% sequence identity. Cluster classification using PhagesDB assigned KRDG1 to cluster K, subcluster K1.

Genome annotation and manual curation using PECAAN identified a total of 95 open reading frames (ORFs). Among these, 39 ORFs were assigned putative functions based on homology (Table 1) while the remaining 56 ORFs were annotated as hypothetical proteins/ no known function. Additionally, one tRNA gene coding for tryptophan (anticodon CCA) was identified. Promoter prediction using the PhagePromoter tool revealed 17 promoter regions, and 19 Rho-independent transcription terminators were identified using the ARNold server. The genes are organized in a modular genome architecture and are described according to their predicted roles in structural assembly, host lysis, replication, regulation, recombination, metabolism, and toxin–antitoxin systems, consistent with the modular organization commonly observed in bacteriophage genomes (Hatfull, 2018).

**Table 1:** List of annotated ORFs in the genome of Mycobacteriophage KRDG1.

S. No.	ORF	Function	Direction	Start	Stop	Length
1	ORF 6	Terminase	+	1,458	2,882	1,425
2	ORF 7	Portal protein	+	2,894	4,447	1,554
3	ORF 8	MuF- like minor capsid protein	+	4,452	6,926	2,475
4	ORF 10	Scaffolding protein	+	7,142	7,675	534
5	ORF 11	Major capsid protein	+	7,750	8,685	936
6	ORF 12	Head-to- tail adaptor	+	8,797	9,183	387
7	ORF 13	Head-to- tail stopper	+	9,183	9,536	354
8	ORF 15	Tail terminator	+	9,795	10,220	426
9	ORF 17	Tail assembly chaperone	+	11,049	11,489	441
10	ORF 19	Tape measure protein	+	11,899	16,053	4,155
11	ORF 20	Minor tail protein	+	16,154	17,290	1,137
12	ORF 21	Minor tail protein	+	17,291	19,057	1767
13	ORF 23	Minor tail protein	+	19,614	20,696	1,083
14	ORF 24	Minor tail protein	+	20,703	21,011	309
15	ORF 25	Minor tail protein	+	21,012	23,444	2,433
16	ORF 26	Minor tail protein	+	23,456	24,490	1,035
17	ORF 29	Lysin A	+	25,222	26,877	1,656
18	ORF 30	Lysin B	+	26,874	27,746	873
19	ORF 31	Holin	+	27,757	28,197	441
20	ORF 34	MRE11 double-strand break endo/exonuclease	+	28,791	29,999	1,209
21	ORF 39	Queuine trna-ribosyltransferase	+	31,601	32,455	855
22	ORF 42	Tyrosine integrase	+	33,177	33,983	807
23	ORF 43	Helix-turn-helix DNA binding domain	+	34,106	34,345	240
24	ORF 44	Excise	+	34,342	34,608	267
25	ORF 46	Membrane protein	+	35,127	35,294	168
26	ORF 48	Helix-turn-helix DNA binding domain, MerR-like	+	35,449	36,240	792
27	ORF 49	WhiB family transcription factor	+	36,237	36,500	264
28	ORF 56	DnaE-like DNA polymerase III	+	38,883	39,437	555
29	ORF 59	Cas4 family endonuclease	+	39,906	40,793	888
30	ORF 65	NrdH-like glutaredoxin	+	42,349	42,591	243
31	ORF 67	HNH endonuclease	+	42,794	43,165	372
32	ORF 68	DNA primase/helicase	+	43,207	45,825	2,619
33	ORF 69	RusA-like resolvase	+	46,237	46,935	699
34	ORF 75	SprT-like protease	+	49,277	49,738	462
35	ORF 78	Membrane protein	+	50,350	51,243	894
36	ORF 84	rtcB-like RNA ligase	+	52,889	54,076	1,188
37	ORF 90	Antitoxin in toxin/antitoxin system, HicB-like	+	56,576	56,773	198
38	ORF 91	HicA-like toxin	+	56,895	57,110	216
39	ORF 95	HNH endonuclease	+	58,409	58,636	228

#### 3.3.1 Structural and assembly proteins

The structural gene module of mycobacteriophage KRDG1 comprises all essential components for virion assembly. The terminase (ORF 6) and portal protein (ORF 7) mediate genome packaging. Head assembly is supported by the MuF-like minor capsid protein (ORF 8), scaffolding protein (ORF 10), and the major capsid protein (ORF 11). Tail formation involves the head-to-tail adaptor (ORF 12), head-to-tail stopper (ORF 13), tail terminator (ORF 15), tail assembly chaperones (ORF 17), and tape measure protein (ORF 19). Six minor tail proteins (ORFs 20, 21, 23–26) likely mediate host recognition and genome delivery as minor tail proteins in mycobacteriophages are known to form tail tip structures involved in host interaction and infection.

#### 3.3.2 Host lysis genes

KRDG1 employs a canonical lysis cassette composed of Lysin A (ORF 29), Lysin B (ORF 30), and holin (ORF 31). Lysin A degrades peptidoglycan, Lysin B targets the mycolic acid-rich outer membrane of *Mycobacterium* species, and holin disrupts the inner membrane to facilitate endolysin access, enabling efficient progeny release at the end of the lytic cycle.

#### 3.3.3 Lysogeny associated genes

Phage KRDG1 encodes hallmark genes associated with lysogeny. A tyrosine integrase (ORF 42) mediates site-specific integration of the phage genome into the host chromosome, while an excise (Xis) (ORF 44) facilitates prophage excision during induction. These genes confirm KRDG1’s potential to establish lysogeny, supporting its classification as a temperate mycobacteriophage.

#### 3.3.4 DNA replication and repair

KRDG1 encodes a DnaE-like DNA polymerase III (ORF 56), a primase/helicase (ORF 68), and several DNA processing enzymes, including an MRE11-like endo/exonuclease (ORF 34), HNH endonucleases (ORF67, ORF95), a Cas4 nuclease (ORF 59), and a RusA-like resolvase (ORF 69). These proteins are likely involved in DNA replication, homologous recombination, and DNA repair during the lytic and lysogenic phases.

Additionally, KRDG1 carries several accessory genes, including queuine tRNA-ribosyltransferase (ORF 39), helix-turn-helix DNA-binding domain proteins (including a MerR-like variant) (ORF 48), a WhiB family transcription factor (ORF 49), NrdH-like glutaredoxin (ORF 65), SprT-like protease (ORF 75), RtcB-like RNA ligase (ORF 84), two predicted membrane proteins (ORF 46, ORF 78), and a HicA–HicB toxin-antitoxin module ORF 90, ORF 91, respectively. While their specific functions in KRDG1 remain uncertain, these genes are present across various sub cluster K1 mycobacteriophages and may be involved in regulatory or auxiliary functions.

#### 3.3.5 Sequence clustering and comparative genomic analysis of KRDG1

Hierarchical clustering based on shared gene content showed that KRDG1 grouped most closely with Kashi_VT1 (ON687735.1) and CrimD (NC_014459.2) among the selected sub cluster K1 mycobacteriophages within the class Caudoviricetes (Figure 2b). These phages shared similar structural, replication, and lysis-associated gene modules. A secondary group of phages including Prithvi (NC_051626.1), TreyKay (MF472892.1), LindT (KX641264.1), Joy99 (MH536822.1), and Atiba (MN234230.1) also showed similarity to KRDG1 but differed in genes related to regulation, integration, and host immunity. Adephagia (NC_051610.1) showed differences in lysogeny-associated genes while retaining similarity in several functional modules. In contrast, Pokerus (ON081329.1), Truffula_tree (OR253900.1), Rapunzel97 (MN234231.1), and Slimphazie (MF140428.1) formed a distinct grouping showing variation in regulatory and integration-associated genes within the K1 subcluster. Additional phages such as Actinup (MH051246.1), HedwigODU (KX585253.1), JAWS (NC_042332.1), Tachez (MF140430.1), Ramen (MN234197.1), and Tiri (ON526984.1) showed intermediate variation in accessory gene content while maintaining similarity in structural and replication-associated genes. This clustering pattern was further supported by average nucleotide identity (ANI) analysis, which revealed high genome-wide similarity between KRDG1 and sub cluster K1 phages, with ANI values ranging from 90.48 to 98.67%, and the highest identity observed with KVT1 (98.67%) while Prithvi, CrimD, and Atiba exhibited ANI values of 92.95, 92.47, and 91.06%, respectively consistent with its closest clustering relationship. Overall, the clustering pattern highlights similarities and differences in functional gene organization among the compared K1 mycobacteriophages.

Comparative genomic alignment of KRDG1 with the closely related K1 phages Kashi_VT1 and CrimD is shown in Figure 2c(ii). The alignment provides an additional genomic comparison within the K1 subcluster.

Comparative genomic analysis of KRDG1 (sub cluster K1) was performed with ZoeJ (KJ510412.1) (sub cluster K2) and Fionnbharth (NC_027365.1) (sub cluster K4), which are Cluster K mycobacteriophages previously employed in phage therapy studies to provide broader comparative context for therapeutic relevance and genome organization. ZoeJ in the treatment of skin infections caused by *M. abscessus*, and Fionnbharth in a mouse model of tuberculosis. (Dedrick et al., 2019b; Smytheman et al., 2025). This comparison reveals both the similar architecture and distinct genomic features of the three Cluster K phages Figure 2c(i). Their genome sizes are 58,681 bp, 57,315 bp, and 58,076 bp, respectively, with GC contents of 66.6, 68.5, and 68.0%, reflecting the typical high GC bias of mycobacteriophages. KRDG1, ZoeJ and Fionnbharth encode 95, 92, 94 predicted genes respectively, indicating comparable gene content. Despite these similarities, specific gene types and their genomic organization distinguish each phage. For instance, KRDG1 and Fionnbharth each carry a single tRNA gene (for tryptophan and lysine, respectively), whereas ZoeJ lacks tRNA genes altogether. Notably, the tRNA gene in KRDG1 is positioned at the left genomic end upstream of the terminase gene, while in Fionnbharth it is located near the genome’s midpoint, just upstream of the integration cassette.

The left arm of the genome encodes genes involved in DNA packaging and assembly. All three phages showed similar synteny in this region. KRDG1 and Fionnbharth possess genes for head-to-tail stopper and tail terminator proteins, which are absent in ZoeJ. While ZoeJ and Fionnbharth each encode two tail assembly chaperones, KRDG1 carries only one. In contrast, KRDG1 encodes six minor tail proteins, ZoeJ eight, and Fionnbharth four. The lysis module comprising lysin A, lysin B and holin is present in all three genomes and is identical in location and function. In terms of accessory genes, KRDG1 encodes an Mre11-like double-stranded DNA endo/exonuclease and a queuine tRNA-ribosyltransferase, both absent in Fionnbharth. ZoeJ shares the latter but also carries a lamD-like protein. The integration cassette, located centrally in all three genomes, contains a tyrosine integrase and excisionase in each, while ZoeJ and Fionnbharth additionally encode an immunity repressor, and ZoeJ uniquely contains a Cro-like regulator, reflecting subtle variations in lysogenic regulation strategies.

The right genomic arm, which encodes replication, recombination, and regulatory genes, showed greater variation in gene content and genomic organization among the three phages compared to the relatively similar structural gene organization observed in the left genomic arm. Shared genes across all three include a DNA polymerase III (either DNAe-like in KRDG1 or DNAq-like in ZoeJ and Fionnbharth), NrdH-like glutaredoxin, DNA primase, RusA-like resolvase, SprT-like protease, and one or more HNH endonucleases. KRDG1 further contains a WhiB family transcription factor, Cas4-family endonuclease, membrane proteins, RtcB-like RNA ligase, and a toxin-antitoxin module composed of HicA-like toxin and HicB-like antitoxin probably derived from the bacterial host during its evolution. ZoeJ uniquely encodes nucleoside deoxyribosyltransferase and two DNA helicases, while Fionnbharth possesses two methyltransferases and shares the Cas4 endonuclease with KRDG1.

KRDG1, ZoeJ, and Fionnbharth share a similar genomic backbone characteristic of cluster K mycobacteriophages, while also exhibiting differences in structural gene content, regulatory modules, accessory genes, and tRNA positioning. These variations highlight genomic diversity and differences in genome organization among K subclusters. Overall, the comparative analysis demonstrates the modular architecture and genomic synteny variation commonly observed among cluster K mycobacteriophages.

### 3.4 Thermal and pH stability test

**Figure 3:**
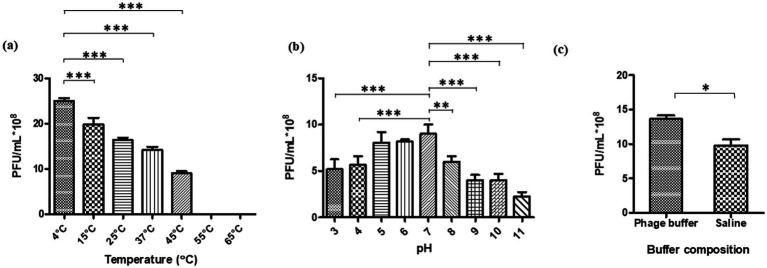
Thermal and pH stability profile of mycobacteriophage KRDG1. **(a)** Thermal stability of KRDG1. The phage exhibited the highest PFU counts at 4 °C and 15 °C. A progressive decline in viability was observed at higher temperatures, with notably reduced titers at 25 °C and 37 °C, and minimal activity remaining at 45 °C. **(b)** pH stability of KRDG1. The phage showed maximum stability at pH 6 and 7 and remained mostly stable at pH 5. Stability decreased at pH 3, 4 and 8 with a sharp decline in infectivity observed at pH 9 to 11. Statistical significance: *p* ≤ 0.01 (**), *p* ≤ 0.0001 (***). Statistical analysis was performed using Dunnett’s Multiple Comparison Test and Graphs were generated using GraphPad Prism software (version 5.01). **(c)** Comparison of KRDG1 titers in phage buffer and saline at pH 7. Phage titers were significantly lower in saline than in phage buffer (EOP = 0.72; paired t-test, *p* = 0.0160).

The thermal stability of KRDG1 was evaluated over a temperature range of 4 °C to 65 °C (Figure 3a). The phage showed the highest stability at 4 °C. A gradual decline in infectivity was observed with increasing temperature, with a 20.8% decrease at 15 °C, 34.4% at 25 °C, and 43.2% at 37 °C. A marked reduction of 63.6% was noted at 45 °C. No detectable phage activity was observed at 55 °C and 65 °C, indicating complete loss of infectivity at these elevated temperatures. These findings suggest that KRDG1 remains stable up to 37 °C but loses viability at elevated temperatures. Statistical analysis using Dunnett’s Multiple Comparison Test revealed statistically significant reduced phage titers at 15 °C, 25 °C, 37 °C, and 45 °C compared to 4 °C (*p* < 0.05), while no significant differences were observed at 55 °C and 65 °C due to total loss of infectivity.

The pH stability of KRDG1 was evaluated across a pH range of 3.0 to 11.0 (Figure 3b). Maximum stability was observed at pH 7. The phage remained highly stable at pH 6 and 5, with only 8.9 and 10.0% reductions in infectivity, respectively, indicating strong stability in the near-neutral pH range of 5–7. In more acidic conditions, infectivity decreased by 36.7% at pH 4 and 42.2% at pH 3. A moderate decline of 33.3% was observed at pH 8. In contrast, alkaline pH values resulted in a marked drop in phage stability, with reductions of 55.6% at both pH 9 and 10, and a substantial 74.4% decrease at pH 11. These findings demonstrate that KRDG1 retains optimal infectivity under near-neutral conditions but shows significantly reduced stability in both strongly acidic and alkaline environments. Statistical analysis using Dunnett’s Multiple Comparison Test revealed significant reduction in phage titers at pH 3, 4, 8, 9, 10, and 11 compared to pH 7 (*p* < 0.05), while no significant differences were observed at pH 5 and 6. To assess the effect of buffer composition, phage titers were determined in saline and phage buffer at pH 7 (Figure 3c). A significant reduction in phage titer was observed in saline compared with phage buffer (EOP = 0.72; paired t-test, *p* = 0.0160).

### 3.5 Host range

**Figure 4:**
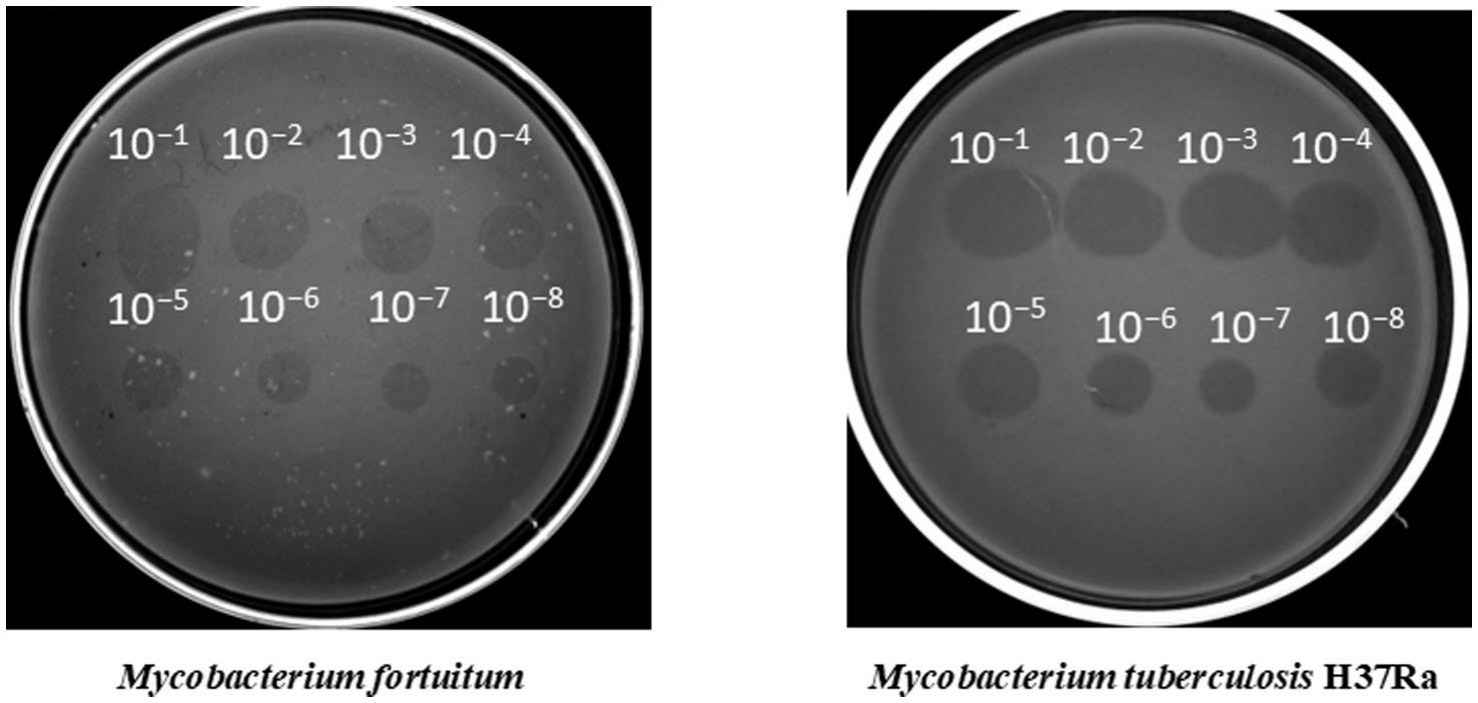
Host range of mycobacteriophage KRDG1 on *Mycobacterium* species. KRDG1 lysate was serially diluted ten-fold (10^−1^–10^−8^), and 3 μL of each dilution was spotted onto top agar overlays containing *Mycobacterium fortuitum* and *Mycobacterium tuberculosis* H37Ra. Zones of lysis/plaque formation were observed on both hosts up to the 10^−8^ dilution, indicating a broad host range of KRDG1 among the tested mycobacterial species.

Phage KRDG1 produced plaques both on *M. fortuitum* and *Mtb* H37Ra up to 10^−8^ dilution (Figure 4). No plaques were observed on *M. marinum* even after 7–14 days of incubation. KRDG1 did not infect any of the non-mycobacterial strains tested (*E. coli, E. faecalis, P. aeruginosa, S. aureus* and *K. pneumonia*) (Table 2). These findings indicate that KRDG1 is highly specific to its mycobacterial hosts, showing a genus-specific broad host-range across different *Mycobacterium* species.

**Table 2:** Host range of phage KRDG1 across different bacterial strains, with infectivity and growth type.

S.no.	Host	Strain	Host growth type	KRDG1 infectivity
1	*Mycobacterium smegmatis*	mc^2^155 (ATCC 700084)	Fast-growing	Infective
2	*Mycobacterium fortuitum*	TMC 1529 (ATCC 6841)	Fast-growing	Infective
3	*Mycobacterium tuberculosis*	H37Ra (ATCC 25177)	Slow-growing	Infective
4	*Mycobacterium marinum*	TMC 1218 (ATCC 927)	Slow-growing	Non-infective
5	*Escherichia coli*	DH5α	Non-mycobacterial	Non-infective
6	*Enterococcus faecalis*	NCTC 775 (ATCC 19433)	Non-mycobacterial	Non-infective
7	*Pseudomonas aeruginosa*	Boston 41,501 (ATCC 27853)	Non-mycobacterial	Non-infective
8	*Staphylococcus aureus*	NCTC 8532 (ATCC 12600)	Non-mycobacterial	Non-infective
9	*Klebsiella pneumoniae*	NCTC 9633 (ATCC 13883)	Non-mycobacterial	Non-infective

### 3.6 Optimal MOI determination for KRDG1

**Figure 5:**
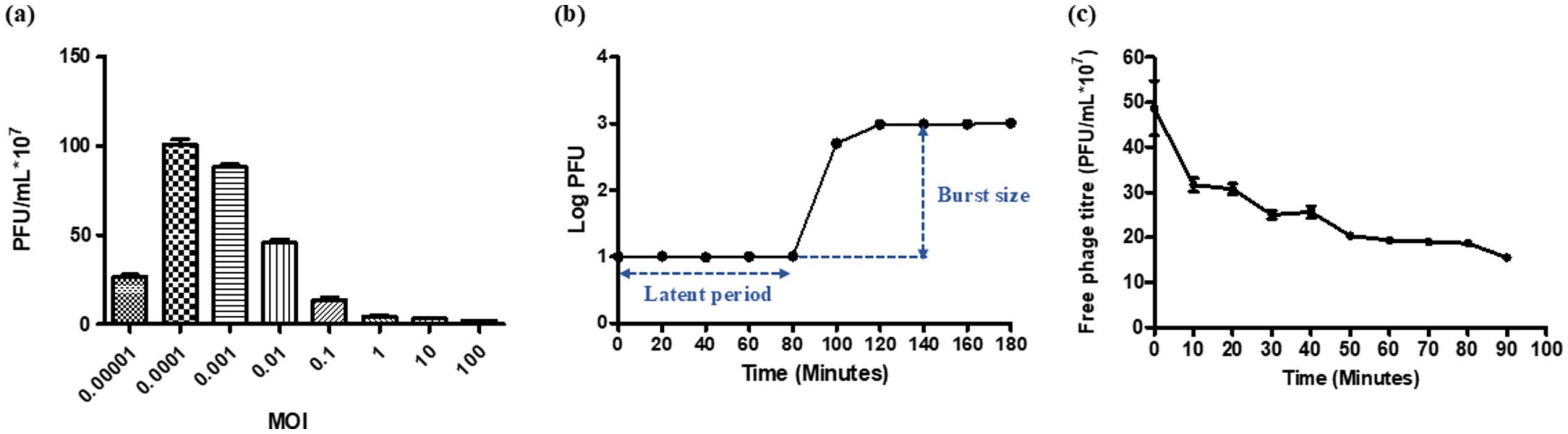
**(a)** Assessment of optimal MOI for KRDG1. The relationship between varying MOI values and resulting phage titers was assessed to determine the most effective infection ratio. The *X*-axis indicates different MOI values, while the *Y*-axis shows the phage titer (PFU/mL*10^7^). The optimal MOI for KRDG1 was identified as 0.0001. **(b)** One step growth assay of mycobacteriophage KRDG1. The *X*-axis represents time (minutes) and the *Y*-axis indicates phage titer in Log PFU. The latent period was 80 minutes with a burst size of 100 phages per bacterial cell. Phage titer reached a plateau at 140 minutes post-infection. **(c)** Adsorption assay of mycobacteriophage KRDG1 to *Mycobacterium smegmatis* mc^2^ 155. Time (minutes) is shown on the *X*-axis, and the unadsorbed (free) phage titre (PFU/mL*10^7^) on the *Y*-axis. A 48.5% reduction in free phages was observed within approximately 30 minutes, indicating efficient adsorption of KRDG1 to host cells. Graphs were generated using GraphPad Prism software (version 5.01).

MOI is calculated as the ratio of PFU/mL to CFU/mL. The highest phage titer (~1.1 × 10^9^ PFU/mL) was observed at an MOI of 0.0001, followed closely by the MOI of 0.001 (Figure 5a). As the MOI increased beyond 0.001, phage titers declined significantly at higher MOIs of 1, 10, and 100. These results indicate that KRDG1 replicates most efficiently at low MOI, where conditions favor lytic growth and higher phage amplification.

### 3.7 One step growth curve

A one-step growth curve assay was performed to assess the infection dynamics of phage KRDG1. The latent period, defined as the interval between phage adsorption and the initiation of progeny release, was 80 min. This was followed by a rise phase, during which phage particles were actively released from lysed host cells (Figure 5b). The burst size, representing the average number of phages produced per infected bacterial cell, was calculated using the ratio of final to initial plaque-forming units (PFU) and was 100 phages per bacterial cell. Phage titer reached a plateau at 140 min post-infection.

### 3.8 Adsorption assay

This assay indicated that KRDG1 adsorption to *M. smegmatis* progressed gradually over time, with about 35% adsorbed by 10 min, 38% by 20 min, 48.5% by 30 min, and 58.2% by 50 min. By 60 min, around 60% of the phages had adsorbed, ultimately reaching approximately 68% by 90 min, where adsorption plateaued indicating a gradual but steady binding to the host over time. (Figure 5c).

### 3.9 Infection kinetics of *M. smegmatis* in response to phage KRDG1 and antibiotics

**Figure 6:**
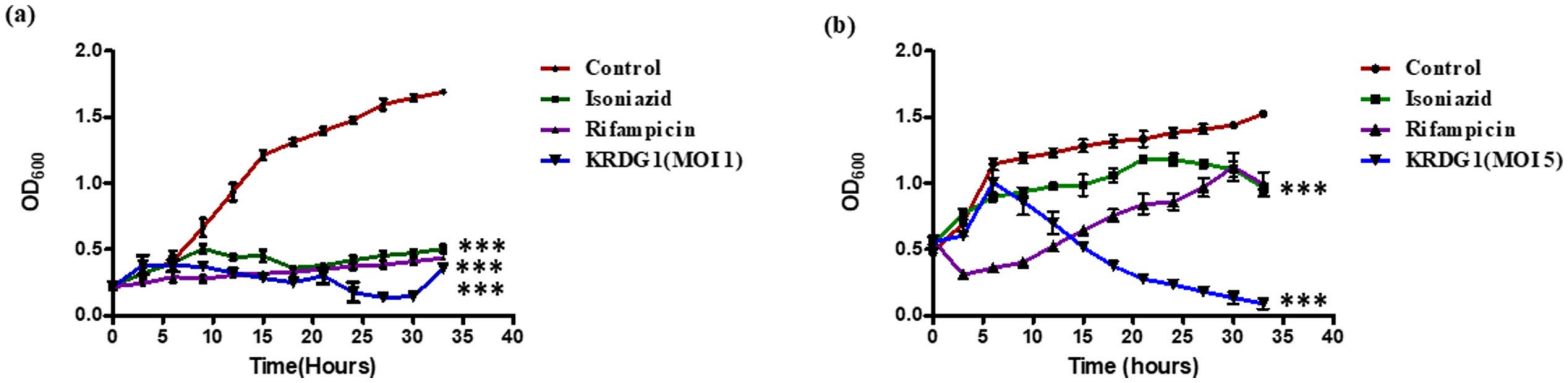
Growth inhibition studies of *M. smegmatis* in liquid culture by phage KRDG1 and antibiotics (isoniazid and rifampicin) during lag and log phase. OD_600_ was measured every 3 h upto 33 h to monitor bacterial growth. The *x*-axis represent time (hours) and the *y* axis indicates optical density at 600 nm (OD_600_). **(a)** Treatment during the lag phase with phage KRDG1 (MOI 1) exhibits comparable inhibition to antibiotics (isoniazid and rifampicin) **(b)** Treatment during the log phase with phage KRDG1 (MOI 5) shows better inhibition when compared to antibiotics (isoniazid and rifampicin). Statistical analysis was performed using Tukey’s Multiple Comparison Test. Graphs were generated using GraphPad Prism software (version 5.01).

The infection kinetics of *M. smegmatis* were assessed following treatment with phage KRDG1 and the antibiotics isoniazid and rifampicin during both the lag phase (OD₆₀₀ ≈ 0.18) and log phase (OD₆₀₀ ≈ 0.5). In the lag phase (Figure 6a), treatment with isoniazid and rifampicin led to significant growth inhibition relative to the control (OD₆₀₀ ≈ 1.65), with rifampicin showing greater efficacy, as OD₆₀₀ values stabilized around 0.4–0.5. Phage KRDG1 at an MOI of 1 also significantly inhibited bacterial growth, resulting in a marked decline in OD₆₀₀ to approximately 0.145 between 15–30 h. A slight increase in OD₆₀₀ toward the 33-h mark may indicate bacterial regrowth. Tukey’s Multiple Comparison Test revealed statistically significant reductions for isoniazid, rifampicin, and KRDG1 (MOI 1) treatments compared to the control (*p* < 0.05).

In the log phase (Figure 6b), isoniazid-treated cultures showed limited growth inhibition (OD₆₀₀ ~ 0.9) compared to the control (~1.5), while rifampicin induced a transient decline in bacterial density followed by regrowth, suggesting initial bactericidal activity with subsequent recovery. Notably, phage KRDG1 at a higher MOI of 5 resulted in a rapid and sustained reduction in OD₆₀₀, dropping below 0.2 by 33 h, confirming enhanced efficacy of phage treatment when administered during the active growth phase. Tukey’s Multiple Comparison Test showed statistically significant reductions in the rifampicin- and KRDG1-treated groups compared to the control (*p* < 0.05), whereas the isoniazid group showed no significant reduction.

Phage KRDG1 was initially applied at MOI 1 to log-phase cultures, but this did not result in significant bacterial killing, likely due to the high host cell density and rapid division during this phase (data not shown). Therefore, the MOI was increased to 5, which led to effective bacterial killing and sustained suppression. This demonstrates the dose-dependent efficacy of phage KRDG1 and the critical influence of bacterial growth phase on infection outcomes.

### 3.10 Fluorescence microscopy

**Figure 7:**
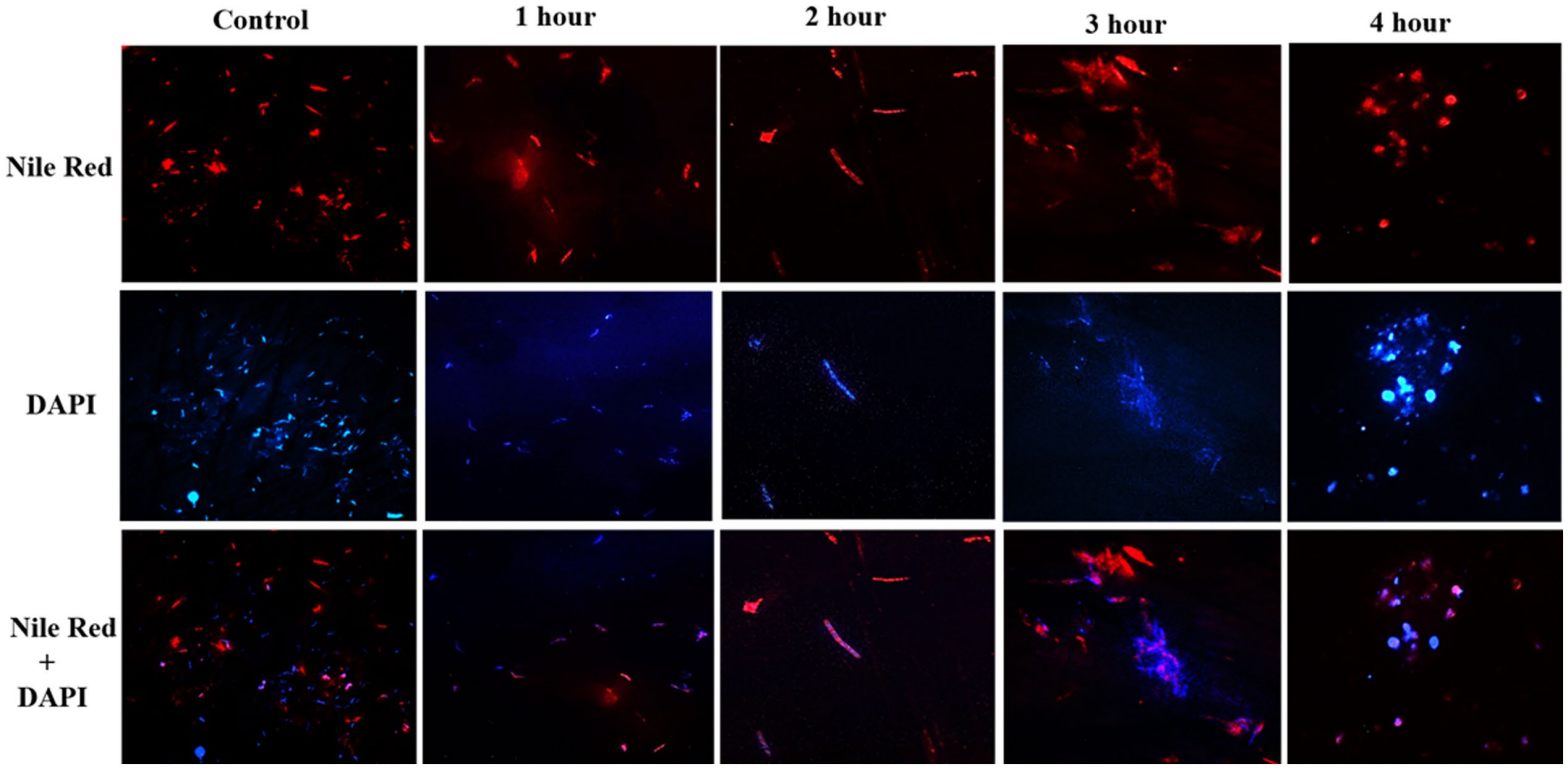
Fluorescence microscopy depicting the lysis of *M. smegmatis* mc^2^155 by incubating with phage KRDG1 at an MOI of 5 for different time points: 1 h, 2 h, 3 h and 4 h. Nile red staining indicates cell membrane and DAPI (blue) indicates nucleoid region. 4 microliter of *M. smeg* phage suspension was placed on an agar pad and bacterial cell lysis and complete blobbing are observed after 3 h of incubation.

Fluorescence microscopy using Nile Red and DAPI staining was employed to visualize the infection dynamics of phage KRDG1 in *M. smegmatis* (Figure 7). In the control samples, strong and uniform fluorescence signal indicated intact cell wall and preserved DNA, respectively. Upon phage infection, noticeable changes in fluorescence patterns were observed over time. While minimal changes were observed at earlier time points, signs of cellular disintegration became evident by 3 h post-infection, with disrupted membrane structures and diffuse/ scattered fluorescent signals of DAPI staining indicating DNA leakage. By 4 h, the majority of bacterial cells had undergone complete lysis, as reflected by the loss of distinct cell morphology and the presence of nuclear blobbing corresponding to fragmented cellular debris. This shows that KRDG1 initiates host cell lysis by 3 h post-infection, leading to complete cellular breakdown by 4 h.

## 4 Discussion

Mycobacteriophage KRDG1 was isolated and characterized to investigate its biological and genomic features. The genome of KRDG1 exhibits the modular and mosaic architecture typical of mycobacteriophages, with synteny across structural, replication, and regulatory regions. With a genome size of 58,681 bp and a GC content of 66.6%, KRDG1 shows high similarity to other sub cluster K1 mycobacteriophages, including KVT1 (Nayak et al., 2023) and CrimD (Pope et al., 2011). The morphological analysis of phage KRDG1 revealed a head (50 nm) and a long non-contractile tail (140 nm), consistent with the siphovirus morphotype commonly observed among mycobacteriophages. Compared to other siphovirus morphotypes, KRDG1 possesses a relatively shorter tail. KVT1 exhibits a tail length of 225 nm (Nayak et al., 2023), while Vic9 and WIVsmall possess tails ranging from 212–265 nm (Zaychikova et al., 2025; Guo et al., 2023). Phage Lang displays a tail length of 189 nm, which is comparatively closer to KRDG1 (Lang et al., 2023). On the other hand, myovirus morphotype with contractile tails are comparatively less common among mycobacteriophages and are largely restricted to Cluster C phages such as KSSH1, which possesses a tail length of 152 nm (Nayak et al., 2025). Podoviral morphotypes have not been reported among mycobacteriophages, possibly due to the complex lipid-rich mycobacterial cell wall, which may hinder successful DNA injection by phages with short tails (Hatfull, 2018). Overall, the siphoviral morphology of KRDG1 is consistent with the predominance of long-tailed mycobacteriophages and may support efficient interaction with the mycobacterial cell envelope.

The genome of mycobacteriophage KRDG1 encodes genes associated with structural and assembly proteins, host lysis functions, lysogeny, and DNA replication and repair, along with several accessory genes. The presence of both a tyrosine integrase and an excisionase supports its temperate nature. While KRDG1 does not encode a classical immunity repressor, which is present in related phages from other subclusters such as ZoeJ (K2) and Fionnbharth (K4), the presence of two helix-turn-helix DNA-binding domain/MerR-like proteins (ORF43 and ORF48) adjacent to the integrase and excisionase suggests the possibility of a novel, uncharacterized repressor with DNA-binding function. This may point to alternative mechanisms of lysogeny regulation. Structurally, KRDG1 encodes only one tail assembly chaperone, in contrast to the two found in ZoeJ and Fionnbharth, and exhibits variation in the number and identity of minor tail proteins. These differences may reflect distinctions in phage assembly or host interaction processes. Additionally, the tRNA gene in KRDG1 is located upstream of the terminase, differing from its position near the integration cassette in Fionnbharth (Guerrero-Bustamante and Hatfull, 2024). Thus, KRDG1 shares a similar genomic backbone with other Cluster K mycobacteriophages, but it also exhibits subcluster-specific features in its structural and regulatory regions.

Although KRDG1 exhibited high nucleotide similarity to the closely related K1 phage KVT1 (99.5% BLASTn identity; 98.67% ANI), several genomic and biological differences were observed. Comparative genomic alignment with KVT1 and the K1 phage CrimD further highlighted differences in genome organization among these closely related phages [Figure 2c(ii)]. KRDG1 possesses a smaller genome (58,681 bp versus 61,010 bp of KVT1), encodes 95 predicted ORFs compared with 101 in KVT1, and produces smaller plaques. In addition, KRDG1 exhibited a lower optimal MOI for amplification, a shorter latent period, distinct pH and temperature stability profiles, and delayed lysis kinetics compared with KVT1. These observations indicate that despite their close genomic relationship, KRDG1 and KVT1 exhibit distinguishable genomic and biological characteristics.

The temperature and pH stability profiles of KRDG1 are generally comparable with those reported for several mycobacteriophages, while also exhibiting distinct stability characteristics. KRDG1 showed peak infectivity at 4 °C and remained viable up to 37 °C, with a sharp decline at 45 °C and complete inactivation at 55 °C and above. Similar thermal sensitivity has been reported for KVT1 and KSSH1, which also lost activity beyond 45–55 °C (Nayak et al., 2023; Nayak et al., 2025). In contrast, WIVsmall and Fulbright displayed comparatively broader thermal tolerance, remaining viable at higher temperatures (60–70 °C) (Guo et al., 2023; Lopez-Davis et al., 2022), whereas Cepens lost viability even at 37 °C (Cantrell, 2019). These differences indicate variability in thermal adaptation among mycobacteriophages.

In terms of pH stability, KRDG1 exhibited optimal infectivity at pH 7 and retained substantial stability within the near-neutral range of pH 5–7, while infectivity declined sharply below pH 4 and above pH 8, with complete inactivation observed at pH 11. This near-neutral stability profile is comparable to Cepens, which remained stable between pH 7–9 (Cantrell, 2019), but narrower than the broader pH tolerance reported for Fulbright (pH 4–9) and WIVsmall (pH 4–11) (Lopez-Davis et al., 2022; Guo et al., 2023). Similarly, KVT1 and KSSH1 exhibited greater alkaline tolerance, remaining stable up to pH 10 with maximum infectivity observed between pH 8–10 (Nayak et al., 2023; Nayak et al., 2025). Saline-based pH-adjusted solutions were used in the pH stability assay because the standard phage buffer showed precipitation under alkaline conditions. A control experiment at pH 7 showed lower PFU values in saline than in phage buffer (EOP = 0.72; *p* = 0.0160), indicating that buffer composition can influence absolute phage titers. Therefore, the observed pH stability profile of KRDG1 reflects phage stability under the saline-based conditions used for the assay. The stability of KRDG1 under physiological temperature and near-neutral pH conditions aligns with environments such as blood, saliva, and wound sites, whereas reduced stability under highly acidic conditions may limit oral application unless protective formulations are employed. Overall, these characteristics support further evaluation of KRDG1 under physiological and host-associated conditions.

Phage KRDG1 forms clear plaques on *M. smegmatis*, *M. fortuitum*, and *Mtb* H37Ra, but not on *M. marinum* or non-mycobacterial strains, resembling the host range reported for several sub cluster K1 phages such as KVT1 (Nayak et al., 2023). Similar infectivity patterns have also been reported for other K1 phages including Adephagia, Anaya, Angelica, and CrimD, which infect *Mtb* strains but exhibit limited infectivity toward *M. marinum* and *M. bovis* BCG (Pope et al., 2011). TM4, a naturally lytic Cluster K phage, exhibits a comparatively broader host range and infects both fast- and slow-growing mycobacteria, including *M. smegmatis*, *M. fortuitum*, *Mtb*, and certain *M. avium* strains, although plaque efficiency varies among substrains. Similarly, ZoeJ (K2) has been reported to infect *Mtb*, *M. bovis* BCG, *M. avium* Va14(O), and *M. interjectum*, although infectivity varies among strains of the same species (Dedrick et al., 2019b). Engineered lytic derivatives of Cluster K phages, including AdephagiaΔ41Δ43, ZoeJΔ45, and FionnbharthΔ45Δ47, have been evaluated in therapeutic studies against mycobacterial infections (Dedrick et al., 2019a; Guerrero-Bustamante et al., 2021). ZoeJΔ45 was included in a phage cocktail used for compassionate treatment of *M. abscessus* infection, resulting in clinical improvement and bacterial clearance (Dedrick et al., 2019a), while FionnbharthΔ45Δ47 demonstrated tuberculocidal activity against *Mtb* strains in preclinical studies (Smytheman et al., 2025; Guerrero-Bustamante et al., 2021). Although KRDG1 infected *M. fortuitum* and *Mtb* H37Ra, phage-mediated growth suppression of these species was not evaluated. Further liquid culture-based studies are required to determine its efficacy against these hosts. Collectively, these observations highlight the diversity in host range among Cluster K mycobacteriophages and place KRDG1 within a group of phages that may be suitable for further investigation following engineering into a lytic derivative.

Phage KRDG1 exhibited the highest titer at an MOI of 0.0001 in plate-based optimization assays, indicating that this MOI was optimal for phage amplification under the tested conditions. Among lytic mycobacteriophages, KSSH1 and Vic9 similarly showed low optimal MOIs of 0.01 (Nayak et al., 2025; Zaychikova et al., 2025), whereas temperate phages such as KVT1, WIVsmall, and Lang demonstrated comparatively higher optimal MOIs ranging from 0.1 to 1.0 in *M. smegmatis* (Nayak et al., 2023; Guo et al., 2023; Lang et al., 2023). Low optimal MOIs of 0.0001 have also been reported for phages infecting other bacterial genera, including *Pseudomonas* phages P1, P9, and P20, as well as the *Citrobacter* phage vB_CbrM_HP1, while *Pseudomonas* phage KSL-1 exhibited an optimal MOI of 0.001 (Hu et al., 2025; Huang et al., 2022; Sun et al., 2012). In temperate phages, infection outcomes depend on MOI and nutritional conditions, with higher MOI often favoring lysogeny and lower MOI favoring the lytic cycle in many systems (Zhang et al., 2022). Erez et al. (2017) demonstrated a peptide-based communication system in which signaling molecules accumulate during infection and, once a threshold is reached, promote lysogeny, although the process remains stochastic. Lysogeny also requires coordinated support from infecting phages (Zeng et al., 2010). Bacterial killing is influenced not only by direct lysis but also by secondary factors such as superoxide radicals, which may become less effective at high MOI due to sequestration by excess phages or increased bacterial resistance (Samaddar et al., 2016). This may explain why KRDG1 showed reduced titers at higher MOIs and optimal amplification at an MOI of 0.0001. However, although MOI 0.0001 was optimal in plate-based assays, preliminary experiments indicated that lower MOIs failed to achieve significant bacterial killing in liquid culture (data not shown). This contrast reflects differences between static surface-based systems and liquid environments, where actively dividing bacteria, dilutional effects, and reduced initial phage-host interactions influence infection dynamics. Thus, the distinction between MOI_input (added ratio) and MOI_actual (effective infections), influenced by adsorption efficiency and host density, becomes critical. Higher MOIs are often required in liquid systems to achieve population-wide lysis (Abedon, 2016). In infection kinetics assays, KRDG1 suppressed bacterial growth during the lag phase at an MOI of 1, reducing OD₆₀₀ within 30 h. However, the same MOI was insufficient during the log phase, requiring an MOI of 5 for sustained killing. This phase-specific difference reflects the impact of bacterial physiology, cell density, replication rate, and metabolic state on phage efficacy. Rapidly growing, dense cultures in the log phase likely lower effective infections, requiring higher phage input for synchronized lysis. Compared to other mycobacteriophages such as D29, KSSH1, TM4, PDRPv, and PDRPxv, which suppressed growth for 40–101 h, KRDG1’s effect was shorter, with regrowth at ~33 h. TM4, though temperate in origin, appears fully adapted to the lytic cycle (Kalapala et al., 2020), whereas KRDG1 may retain temperate features limiting lytic persistence. Consistent with previous reports, isoniazid and rifampicin were more effective during the lag phase, while reduced inhibition during the log phase likely reflects decreased antibiotic efficacy in dense, metabolically active bacterial populations (Kalapala et al., 2020). In comparison, phage KRDG1 showed lag-phase activity comparable to that of isoniazid and rifampicin, while exhibiting a more sustained decline in bacterial growth during the log phase at MOI 5. These observations further demonstrate that the MOI optimal for phage amplification may differ from that required for efficient bacterial killing in liquid culture, where higher MOIs improve infection coverage and coordinated lysis. Collectively, these findings highlight the importance of bacterial physiological state, environmental conditions, and effective infection dynamics in determining phage activity. It would be interesting to evaluate whether engineering KRDG1 into a strictly lytic derivative through deletion of integrase and putative repressor-associated genes improves its killing efficiency.

Adsorption is a key step in phage infection, influencing how quickly phages engage with host cells. KRDG1 shows moderate adsorption, with approximately 48.5% binding by 30 min and around 68% by 90 min. This gradual increase suggests that KRDG1 interacts progressively with its host over time. Other lytic mycobacteriophage like Vic9 (belonging to sub cluster B2), exhibits almost complete adsorption by approximately 30 min (Zaychikova et al., 2025) indicating better adsorption in comparison to KRDG1.

The one-step growth analysis of mycobacteriophage KRDG1 revealed a latent period of 80 min and a burst size of 100 PFU per infected cell, indicating efficient replication dynamics. Compared with other temperate mycobacteriophages, KRDG1 exhibited a slightly shorter latent period than KVT1 (90 min, 102 PFU/cell) while maintaining a comparable burst size, and showed substantially faster lysis and higher progeny yield than WIVsmall (120 min, 12.8 PFU/cell) (Nayak et al., 2023; Guo et al., 2023). D29 also shows efficient lysis (60 min), whereas its holin-deficient mutant exhibits delayed lysis (90 min), highlighting the role of holin function in determining the timing of host cell lysis (Lopez-Davis et al., 2022). Among lytic mycobacteriophages, KSSH1 exhibited a shorter latent period and higher burst size (60 min, 200 PFU/cell), while Lang demonstrated even faster lysis kinetics (30–60 min), reflecting replication strategies optimized for rapid host exploitation (Nayak et al., 2025; Lang et al., 2023). These findings align with theoretical models which propose that short latent periods (SLPs) are favored in high bacterial density environments, enabling faster replication cycles at the cost of reduced burst size. Conversely, long latent periods (LLPs) are more advantageous in low-density environments, where maximizing progeny per infection is critical (Abedon et al., 2003). KRDG1’s combination of a moderately short latent period and robust burst size suggests a strategic balance, enabling successful replication under conditions of moderate to high host availability.

Fluorescence microscopy with Nile Red and DAPI staining showed that phage KRDG1 triggers delayed lysis in *M. smegmatis* compared to KSSH1 (Nayak et al., 2025) and KVT1 (Nayak et al., 2023). Membrane damage caused by KRDG1 became evident only after 3 h post-infection, with complete lysis occurring by 4 h, whereas KSSH1 and KVT1 induced host cell lysis within 1–2 h. These differences may reflect phage-specific variations in infection kinetics, including adsorption efficiency, latent periods and lysis regulation. KSSH1, being a virulent phage, exhibited rapid lysis consistent with its short latent period and high burst size, while KVT1 lysed faster than KRDG1 despite being temperate, possibly reflecting differences in efficiency of lytic gene expression. The relatively slower lysis kinetics of KRDG1 may indicate delayed activation of lysis-associated modules rather than a direct increase in lysogenic control. Lysis is a tightly regulated, time-sensitive process involving coordinated action of multiple phage proteins along with virion assembly, and it’s timing critically influences phage reproductive output (Young, 2014).

Therefore, KRDG1 exhibits distinct genomic features, infectivity against multiple mycobacterial hosts, and unique infection dynamics. Its temperate nature suggests potential for engineering to enhance efficacy, while its stability and host specificity support its relevance for further evaluation against *Mycobacterium* species.

## 5 Conclusion

Phage therapy is emerging as a promising alternative to conventional antibiotics in the treatment of tuberculosis (TB) and other mycobacterial infections in the context of increasing drug resistance in *Mtb*. This study presents a comprehensive characterization of Mycobacteriophage KRDG1, a novel temperate phage belonging to Cluster K, capable of infecting *M. fortuitum* and *Mtb* H37Ra beside *M. smegmatis*. A large proportion of mycobacteriophages are temperate and their therapeutic potential is constrained by the risk of lysogeny which can interfere with effective bacterial clearance. However, advances in genetic engineering such as BRED and CRISPR-Cas-based genome editing have enabled the modification of temperate phages into lytic forms suitable for therapeutic use (Kakkar et al., 2024). This study fills critical knowledge gaps by detailing the morphology, genome architecture, and infection kinetics of KRDG1. These insights expand the current understanding of mycobacteriophage biology and help identify phages that may be engineered for clinical applications. The infection dynamics of KRDG1 in liquid cultures under lag and log conditions at different MOIs suggest that genetic engineering with respect to its lysogeny genes and conversion to exclusively lytic lifecycle may improve its suitability for therapeutic and diagnostic applications. This is consistent with recent work on other Cluster K phages like ZoeJ and Fionnbharth, where removal of lysogeny-related genes enabled the generation of lytic phages that were subsequently applied in therapy (Dedrick et al., 2019a). In the context of rising multidrug-resistant mycobacterial infections, systematic characterization of such phages holds significant clinical relevance. Overall, this study provides new insights into the functional and genomic features of Cluster K mycobacteriophages and highlights the underexplored potential of temperate phages. It provides a valuable foundation for future therapeutic development targeting pathogenic mycobacterial and non-mycobacterial species.

## Data Availability

The complete genome sequence of mycobacteriophage KRDG1 has been deposited in GenBank under the accession number ON687736.1. The datasets generated and analyzed during the current study, including temperature and pH stability assays, multiplicity of infection (MOI), adsorption curves, infection kinetics of M. smegmatis (with antibiotic and KRDG1 separately), fluorescence microscopy images, and host range data are available in the Zenodo repository https://zenodo.org/records/16946808 (Gupta et al., 2025) under a Creative Commons Attribution 4.0 International (CC-BY 4.0) license.

## Footnotes

1
https://phagesdb.org/

2
https://cpt.tamu.edu

3
https://phagescope.org

4
https://www.ezbiocloud.net/tools/ani
